# Smartwatch Technology in Medicine: A Call for Future Dermatologic Research

**DOI:** 10.2196/47252

**Published:** 2023-10-16

**Authors:** Emelie E Nelson, Morgan A Rousseau, T Austin Black, Mariya N George, Rashid M Rashid

**Affiliations:** 1 McGovern Medical School at UTHealth Houston Houston, TX United States; 2 Mosaic Dermatology Houston, TX United States

**Keywords:** digital health, dermatology, smartwatch, ultraviolet radiation, ultraviolet, UV, skin cancer, pruritus, sunscreen, device, support, patient education, clinical management, cardiovascular, cancer prevention, prevention, cancer, technology, wearable technology

## Introduction

The use of smartwatches and other wearable devices has been increasingly empowering users with the ability to monitor numerous critical aspects of their health. From monitoring heart rate and blood pressure to detecting arrhythmias, seizure activity, and falls, noninvasive smartwatch technology has proven to be an effective screening tool that can be used to inform patient care and improve outcomes [1]. Additionally, smartwatches are highly portable, relatively affordable, and adequately available to the public, making them an attractive investment for consumers.

The application of smartwatch technology to the field of dermatology has not been well described; however, smartwatch technology could greatly aid in both risk assessment and prevention of skin cancer. This paper examines smartwatch-associated research across all medical specialties and proposes future applications to dermatology, specifically for skin cancer prevention and intervention.

## Methods

A review of the use of smartwatches across all medical specialties was performed. The search terms “smart watch” and “smartwatch” were searched in PubMed for English-language articles published from database inception to April 10, 2023. Multimedia Appendix 1 summarizes the inclusion and exclusion criteria. One reviewer (MAR) screened all articles for inclusion. Studies that satisfied the inclusion and exclusion criteria were included for data extraction. Two reviewers (MAR and TAB) independently performed the full-text review and data extraction, with the primary variable of interest being the medical specialty associated with each article.

## Results

Of the 1333 identified articles, 346 met the study eligibility criteria. Multimedia Appendix 2 displays the frequency of each medical specialty represented. The majority of studies examined smartwatches in the context of cardiovascular research (174/346, 50.4%). Neurology was represented in 15.1% (52/346) of the studies, and the remaining 34.5% (120/346) of studies were distributed across 12 other specialties.

Only 3 studies (<1%) represented dermatologic research (Table 1). One used wrist actigraphy to measure nocturnal scratching in patients with pruritus [2]. The second, by Jang et al [3], measured sleep duration and its impact on skin characteristics in women. Finally, Dey et al [4] used smartwatches to track cumulative UV exposure in patients.

**Table 1:** Summary of dermatology-related smartwatch studies.

Article (author, year, journal)	Methods	Feature of watch used	Outcome studied	Key findings	Smartwatch used
Dey et al [4], 2017, *Eng Med Biol Soc*	Integration of UV sensors into 1200 smartwatches and smartphones	UV exposure	Cumulative UV tracking	Integration of UV sensors into these devices provided an accurate estimate of cumulative UV exposure	Android
Ikoma et al [2], 2019, *Acta Derm Venereol*	Creation of a smartwatch app to detect nocturnal scratching using accelerometer data	Wrist actigraphy	Nocturnal scratching in patients with pruritus	High reliability and clinical usefulness of the newly created app was demonstrated	Apple
Jang et al [3], 2020, *Skin Res Technol*	Already existing sleep-tracking capabilities in smartwatches were used and longitudinally compared to the characteristics of skin aging among participants	Sleep time monitoring	Skin characteristics in women	Negative changes were seen in the skin characteristics of patients who averaged less sleep	Xiaomi

## Discussion

### Principal Findings

Great disparities exist in the use of smartwatch technology across various medical specialties. We propose this is in part due to the specialty-specific capabilities found within smartwatches. For example, the majority of included studies examined applications of smartwatch technology in cardiology, likely due to the device’s ability to measure pulse and respiration rate and perform electrocardiograms [1].

As smartwatches sit on the skin and are thus exposed to the same environmental factors as the wearer, they represent a valuable opportunity to better understand both the UV and non-UV environmental, occupational, and avocational exposures that may contribute to the development of skin cancer. With the incidence of both melanoma and keratinocyte carcinomas continuously increasing [5], understanding the risk factors for the development of skin cancer becomes important for determining individual patient risk, early detection, and improving clinical outcomes. Furthermore, because smartwatches provide continuous monitoring capabilities, personalized alerts could be implemented to notify users of behavioral changes they could employ to reduce the risk of developing skin cancer (ie, “Your UV exposure over the last 7 days is higher than normal. To minimize cancer risk, ensure proper UV protection.”). Use of these continuous monitoring capabilities could be further applied to advance research within the field, allowing for minimally invasive yet highly accurate data collection, which can aid in the development of personalized treatment plans.

Smartwatch technology continues to be refined and improved to better meet the health care needs of consumers. This is perhaps best exemplified by the development of smartwatch-based oxygen saturation measurement capabilities during the COVID-19 pandemic. We propose that future smartwatches be equipped with the technology to measure UV-A and UV-B rays, time spent in water, and air quality, as well as prompt users to reapply sunscreen at regular intervals. The benefits of these implementations are summarized in Table 2.

The benefits of smartwatch technology in skin cancer prevention and intervention are numerous. However, it must be acknowledged that smartwatches can be costly and not accessible to everyone. As such, the quantifiable and generalizable impact of this technology may be somewhat diminished.

**Table 2:** Dermatological applications and benefits of smartwatches.

Intervention	Mechanism	Effect	Special populations of benefit
UV sensor and sunscreen reminder	Provide individuals with a quantitative, cumulative estimate of UV exposure Remind individuals at appropriate intervals to reapply sunscreen	Encourage individuals to reapply sunscreen at regular intervals and to limit time spent outdoors during high UV-index hours	Patients with xeroderma pigmentosum, porphyrias, photoallergy, lupus erythematosus, and other photosensitivity disorders Individuals who are occupationally or recreationally exposed to the sun
Time spent in water monitor	Provide individuals with quantitative estimates of total time spent in water Remind individuals at appropriate intervals to reapply sunscreen	Encourage reapplication of sunscreen	Patients with conditions exacerbated by water such as aquagenic keratoderma Swimmers, surfers, and divers
Air quality	Alert individuals to chemical hazards, pollen levels, or other irritating substances in the atmosphere	Promote the use of protective clothing, sunscreen with topical antioxidants, and the usage of indoor air purifiers or ventilators	Individuals with atopic conditions

### Conclusion

A significant gap in the medical literature exists surrounding the potential uses of smartwatches in the field of dermatology. Nonetheless, the application of smartwatches within dermatology represents a point of meaningful implication, especially as it relates to skin cancer prevention and intervention. As such, future research on smartwatch technology in dermatology is warranted.

## Appendix

Multimedia Appendix 1 Inclusion and exclusion criteria for study eligibility.

Multimedia Appendix 2 Representation of smartwatch-related clinical research among all medical specialties.
