# Supplementary material for: Smartwatch Technology in Medicine: A Call for Future Dermatologic Research
Source: JMIR Dermatol. 2023 Oct 16;6:e47252. doi: 10.2196/47252 (PMC10616727; doi:10.2196/47252)
Supplement: Multimedia Appendix 1 [file derma_v6i1e47252_app1.docx]

**Multimedia Appendix 1.** Inclusion and exclusion criteria for study eligibility.

| **Inclusion Criteria** | **Exclusion Criteria** |
| --- | --- |
| - Articles published in the English language - Use of a smartwatch in patient care - All medical specialties | - Validation studies - Studies unrelated to direct patient care - Technical and machine learning studies |
