# Supplementary figures and images for: Smartwatch Technology in Medicine: A Call for Future Dermatologic Research
Source: JMIR Dermatol. 2023 Oct 16;6:e47252. doi: 10.2196/47252 (PMC10616727; doi:10.2196/47252)

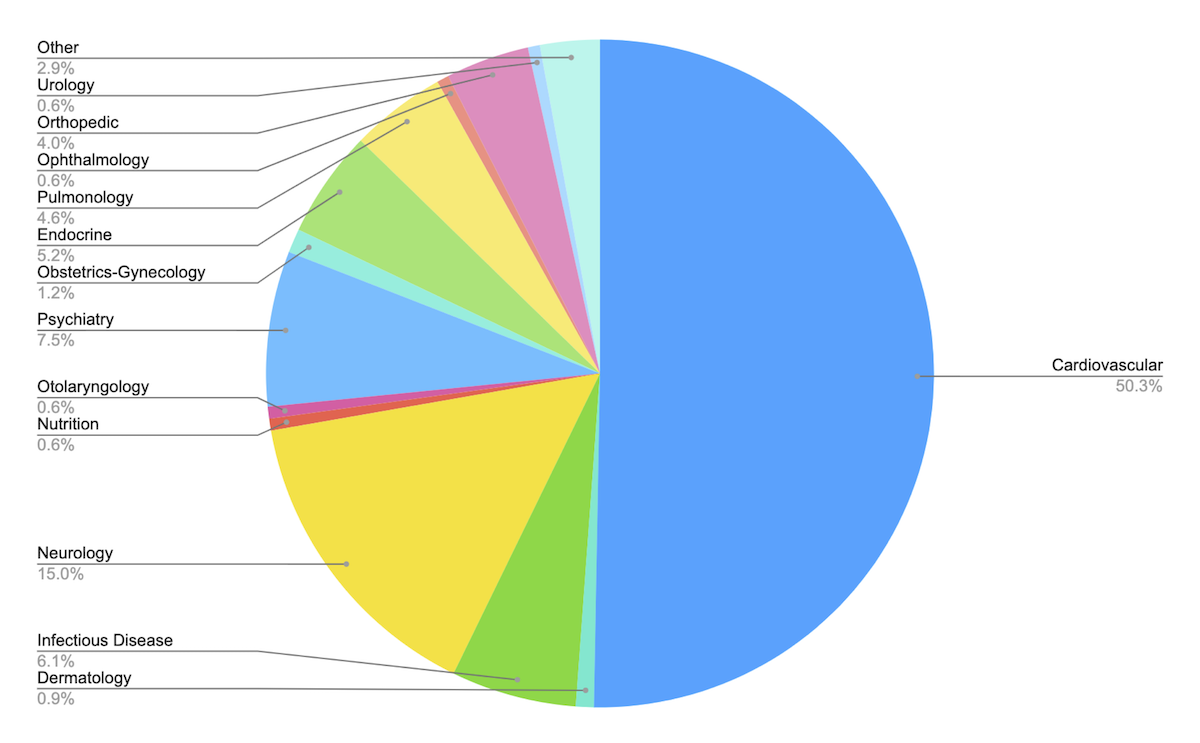

Supplement: Multimedia Appendix 2 [file derma_v6i1e47252_app2.png]
